# Lung Cancer and Vehicle Exhaust in Trucking Industry Workers

**DOI:** 10.1289/ehp.11293

**Published:** 2008-05-30

**Authors:** Eric Garshick, Francine Laden, Jaime E. Hart, Bernard Rosner, Mary E. Davis, Ellen A. Eisen, Thomas J. Smith

**Affiliations:** 1 Pulmonary and Critical Care Medicine Section, Medical Service, Veterans Affairs Boston Healthcare System, Boston, Massachusetts, USA; 2 Channing Laboratory, Department of Medicine, Brigham and Women’s Hospital, Harvard Medical School, Boston, Massachusetts, USA; 3 Exposure, Epidemiology, and Risk Program, Department of Environmental Health; 4 Department of Epidemiology, Harvard School of Public Health, Boston, Massachusetts, USA; 5 School of Economics, University of Maine, Orono, Maine, USA; 6 Environmental and Occupational Medicine and Epidemiology Program, Department of Environmental Health, Harvard School of Public Health, Boston, Massachusetts, USA; 7 Environmental Health Sciences Division, School of Public Health, University of California, Berkeley, California, USA

**Keywords:** diesel exhaust, lung cancer, occupational exposure, particulate matter, traffic

## Abstract

**Background:**

An elevated risk of lung cancer in truck drivers has been attributed to diesel exhaust exposure. Interpretation of these studies specifically implicating diesel exhaust as a carcinogen has been limited because of limited exposure measurements and lack of work records relating job title to exposure-related job duties.

**Objectives:**

We established a large retrospective cohort of trucking company workers to assess the association of lung cancer mortality and measures of vehicle exhaust exposure.

**Methods:**

Work records were obtained for 31,135 male workers employed in the unionized U.S. trucking industry in 1985. We assessed lung cancer mortality through 2000 using the National Death Index, and we used an industrial hygiene review and current exposure measurements to identify jobs associated with current and historical use of diesel-, gas-, and propane-powered vehicles. We indirectly adjusted for cigarette smoking based on an industry survey.

**Results:**

Adjusting for age and a healthy-worker survivor effect, lung cancer hazard ratios were elevated in workers with jobs associated with regular exposure to vehicle exhaust. Mortality risk increased linearly with years of employment and was similar across job categories despite different current and historical patterns of exhaust-related particulate matter from diesel trucks, city and highway traffic, and loading dock operations. Smoking behavior did not explain variations in lung cancer risk.

**Conclusions:**

Trucking industry workers who have had regular exposure to vehicle exhaust from diesel and other types of vehicles on highways, city streets, and loading docks have an elevated risk of lung cancer with increasing years of work.

Diesel exhaust is a complex mixture of particulate matter (PM) and gases and includes particles ≤ 1.0 μm diameter (PM1) with mutagenic and polycyclic aromatic hydrocarbon (PAH) carcinogenic compounds adsorbed to a carbon core and ultrafine particles made up of condensed organics (Kittelson et al. 2002). Approximately 40 epidemiologic studies have described an association between lung cancer risk and occupations with some degree of diesel exhaust exposure, including railroad workers, construction workers, port workers, and truck and other professional drivers (Bhatia et al. 1998; Diesel Working Group 1995; Lipsett and Campleman 1999; Office of Research and Development 2002). However, this association has been questioned (Bunn et al. 2004; Hesterberg et al. 2006; Valberg and Watson 2000) because of uncertainties regarding the link between the occupational records used to assess work history, specific job duties, and exposure. In particular, the likelihood that truck drivers and other workers in trucking industry jobs were exposed to diesel exhaust depends on job duties and historical driving patterns. Previous studies in the trucking industry have lacked detailed work records identifying specific trucking industry–related jobs and had a limited ability to assess job-related exposure differences.

To address these uncertainties, we conducted a retrospective assessment of lung cancer mortality of workers employed by four large unionized (Teamster) carriers. In this study, we used the detailed work records available in this industry to describe the relationship with years of work in specific trucking industry jobs that have different current and historical exposure patterns. We have previously identified elevated standardized mortality ratios (SMRs) for lung cancer mortality in this cohort (Laden et al. 2007). In this industry, the association between job and specific exposure-related duties has remained stable over time, and the dates of diesel and other equipment use are known (Garshick et al. 2002). We conducted an industrial hygiene job review to identify specific jobs and job duties in the industry and the use of diesel and nondiesel vehicles, and we conducted a national exposure assessment to characterize exposures to elemental carbon (EC) and organic carbon (OC) as a marker of exposure to diesel and other traffic-related PM among jobs (Davis et al. 2006, 2007; Smith et al. 2006), including a source apportionment assessment (Sheesley et al. 2008a, 2008b).

## Materials and Methods

### Population

Details of the cohort have been desdribed previously (Laden et al. 2007). We obtained detailed computerized work records for unionized employees employed in 1985 (54,319 men and 4,007 women) from four large national companies. We obtained cause of death from 1985 through 2000 from the National Death Index (National Center for Health Statistics, Hyattsville, MD) to identify deaths with primary lung cancer {*International Classification of Diseases, Ninth Revision,* code 162 [World Health Organization (WHO) 1978] and *International Classification of Diseases, Tenth Revision*, codes C33–C34 (WHO 1993)} anywhere on the death certificate. Lung cancer usually results in death within months to several years, and there was little change in survival rates from 1985 through 2000 (American Lung Association 2005), so it is appropriate to include all lung cancers identified on death certificates as cases. Because 96% of the lung cancer cases occurred in men ≥ 40 years of age in 1985, we limited analyses to these men with at least 1 year of work in a trucking industry job (*n* = 31,135). Job title and duties are the same across companies, and the work records included start date, retirement, and layoff dates that allowed calculation of years of work in each specific trucking industry job. In one company, computerized work records started in 1972, and we calculated work duration from date of hire until 1972 based on the first available job. However, this lack of detailed records represented only 1.5% of the overall years of trucking industry employment. Study approval was obtained from our institutional review boards.

### Exposure assessment

We categorized exposure into eight job categories based on a review of job titles and duties, including a 2001–2005 industrial hygiene exposure assessment (Davis et al. 2006, 2007; Smith et al. 2006) and information on the historical use of diesel and other vehicles by workers in the cohort (Garshick et al. 2002). Table 1 summarizes the number of workers in each job in 1985, along with a description of job duties. We calculated time-varying cumulative years of work in each of the eight job categories. Each worker could potentially accumulate exposure in each job category throughout his career.

### Statistical methods

We used proportional hazard regression to examine relationships between lung cancer mortality and employment duration in each trucking industry job category. We included all eight job-specific exposure variables in regression models to adjust lung cancer risk for different jobs held throughout a worker’s career. We used penalized splines (Thurston et al. 2002) to allow for nonlinearity in mortality risk with employment duration, and calculated the change in hazard ratio (HR) and 95% confidence intervals (CIs) associated with each year of work. To closely adjust for age and lung cancer secular trends, risk sets were generated using attained age in 1-year increments as the timeline, with separate baseline hazards based on decade of age at entry, calendar year, and decade of hire. We considered race, census region of residence (based on last home address), and company as potential confounders in the regression models. Time-varying variables for years employed and years off work were used to adjust for a healthy-worker survivor effect. We performed all analyses in SAS, version 9 (SAS Institute Inc., Cary, NC) or R version 2.3.1 (R Foundation for Statistical Computing, Vienna, Austria).

### Smoking assessment

To assess job-related variation in smoking habits, in 2003 a questionnaire was mailed to a sample of 11,986 active and retired workers in the industry (Jain et al. 2006). The response rate was 40.5%, and distributions of job titles, gender, region of residence, and terminal size and location among responders and nonresponders were similar. We used proportions of current, former, and never-smokers among male Caucasian respondents for each job group to weight cigarette smoking literature-based lung cancer relative risks (RRs) among U.S. men [current smoker, RR = 20.3; former smoker, RR = 10.6 (Thun et al. 1997; U.S. Department of Health and Human Services 1990)]. We calculated a job-specific smoking adjustment factor by dividing the weighted risk for workers employed in each job by the weighted risk for workers not employed in that job (Axelson 1980; Schlesselman 1978) and calculated 95% CIs by considering sampling error in calculating the adjustment factors (Larkin et al. 2000). Additional details are presented in Supplemental Material online (http://www.ehponline.org/members/2008/11293/suppl.pdf).

## Results

### Population

Characteristics of the cohort are presented in Table 2. On average, workers were hired in their mid-30s, most likely due to hiring policies requiring previous experience. Most workers were hired after long-haul (LH) trucks changed from gas to diesel during the 1950s and 1960s, but before or during the transition of pickup/delivery (P&D) trucks from gas to diesel during the 1970s and 1980s. Diesel forklifts were also used by dockworkers on some terminal loading docks during the mid-1980s. Previously, liquefied petroleum gas (propane) and some gasoline forklifts were used, and in the early 1990s, diesel forklifts were phased out and replaced with propane units.

Participants were predominantly Caucasian, lived in the South or Midwest, and worked an average of nearly 22 years, with 73% working between 20 and 40 years. Although jobs in this industry are generally stable, the degree of job stability varied by occupational title; specifically, 85% of LH drivers, 40% of P&D drivers, 52% of combination workers, and 43% of dockworkers remained in the same job from hire through 2000. Most of the switching was between the P&D, dock-worker, and combination groups, and 10% of the LH drivers also worked at one time in P&D or combination jobs.

### Lung cancer mortality

There were 4,306 deaths and 779 cases of lung cancer (734 where lung cancer was the underlying cause) during the follow-up period. Census region and race were significantly associated with lung cancer risk. Compared with the HR for residence in the Northeast or unknown regions, the HR for residence in the South was 1.46 (95% CI, 1.16–1.83), in the Midwest, 1.48 (95% CI, 1.15–1.82), and in the West, 1.17 (95% CI, 0.89–1.53). Caucasians had an HR of 1.49 (95% CI, 1.17–1.88) compared with other races. Company was not a significant covariate.

**Figure 1:**
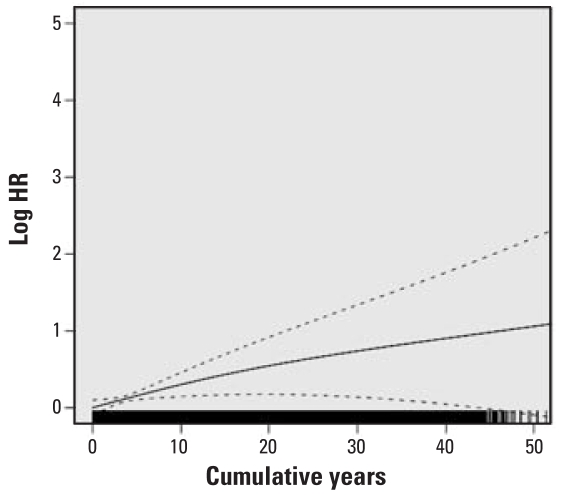
Natural log of the HR and 95% CI for lung cancer risk for cumulative years of work as an LH driver; a histogram of years as an LH driver is at the bottom of the graph.

**Figure 2:**
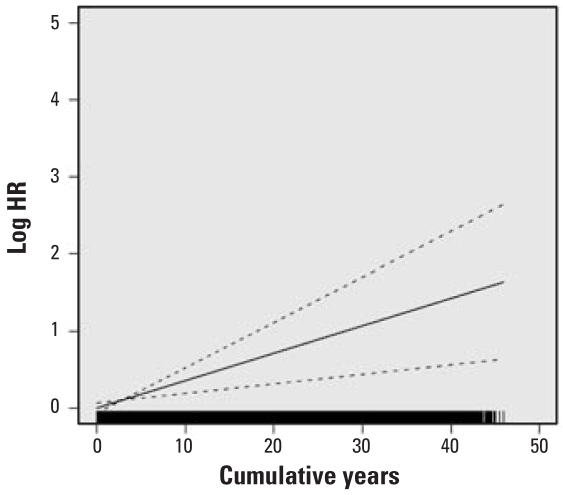
Natural log of the HR and 95% CI or lung cancer for cumulative years of work as a P&D driver; a histogram of years as a P&D driver is at the bottom of the graph.

**Figure 3:**
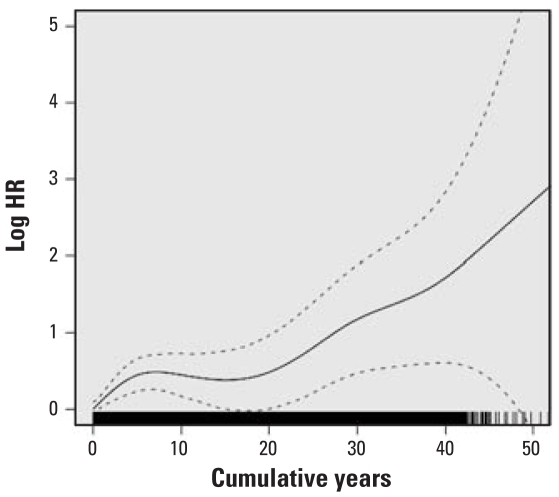
Natural log of the HR and 95% CI for lung cancer for cumulative years of work as a dockworker; a histogram of years as a dockworker is at the bottom of the graph.

**Figure 4:**
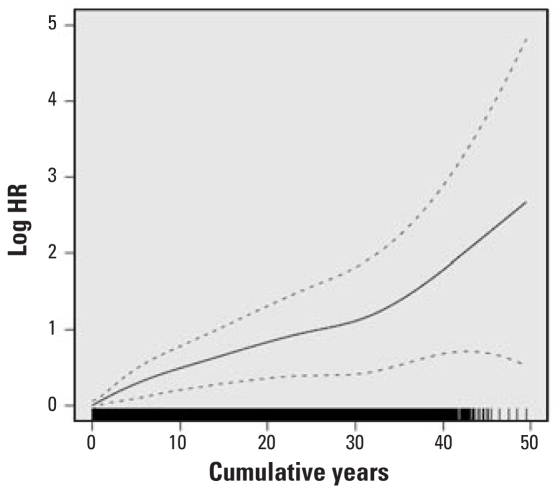
Natural log of the HR and 95% CI for lung cancer for cumulative years of work as a combination worker; a histogram of years as a combination worker is at the bottom of the graph.

HRs for lung cancer mortality with at least 1 year of work in each specific job category, adjusted for years on work, years off work, race, and census region are presented in Table 3. HRs were statistically significantly elevated for dockworkers and combination workers and were elevated but of borderline significance for LH and P&D drivers. HRs were not elevated for work as a hostler or mechanic or for other jobs, and the HR for clerks was statistically significantly reduced. We calculated percent change in the HR per year worked for LH drivers, P&D drivers, dockworkers, and combination workers (Table 4). Although in some jobs (particularly the dockworkers) the increase in risk appeared not to be entirely linear, the increase with years of work did not statistically significantly depart from linearity (Figures 1–4). The change in risk per year of work was similar among P&D drivers, dockworkers, and combination workers (3.4–4.0%), but it was slightly less in LH drivers (2.5%). There were too few lung cancer cases in the other job categories to model years of work reliably, and indicator variables were included for any work in these jobs, as in the model presented in Table 3.

We used job-specific coefficients from the regression models in Table 4 to estimate RR for 20 years of work and calculated smoking adjustment factors to estimate smoking-adjusted risks by dividing each job-specific regression coefficient by an adjustment factor. LH drivers had a smoking adjustment factor > 1, indicating greater smoking rates compared with workers not in that job, whereas the adjustment factors for P&D drivers, dockworkers, and combination workers ranged from 0.92 to 0.96, indicating slightly lower smoking rates compared with other workers. After adjustment, the HR decreased in LH drivers, and the HRs for P&D drivers, dockworkers, and combination workers did not appreciably change and were similar to each other.

## Discussion

We performed a retrospective assessment of lung cancer mortality in trucking industry workers 1985–2000 and found that lung cancer mortality risk was positively associated with years of work in jobs associated with regular exposure to freshly emitted vehicle exhaust. These included jobs with current and historical exposure from driving in cities and on urban highways (P&D drivers, combination workers) and on terminal loading docks with exposure reflecting movement of freight and vehicles in the yard around the terminal, background levels, and forklift exhaust (combination workers, dockworkers). In LH drivers, who drive primarily on intercity highways, the risk per year of work was also elevated, but after adjustment for differences in smoking, the increase in risk did not achieve conventional levels of statistical significance. Workers in other jobs in the cohort had too few lung cancer cases to reliably model lung cancer risk based on years of work. Based on exposure defined as at least 1 year of work, lung cancer risk in the clerks was statistically significantly reduced and the risk in mechanics, hostlers, and workers in other jobs was not significantly elevated.

Our analytic approach has the advantage of using internal cohort-based reference groups (i.e., trucking industry workers in the same cohort) to account for unmeasured factors related to lung cancer mortality. For example, the reference group for LH drivers was all workers who never served as an LH driver, and the reference group for the P&D drivers was all workers who had never worked as a P&D driver. Applying job-specific smoking adjustment factors to account for variation in smoking patterns between each job and reference group as in Table 4 produced results similar to unadjusted risks. The pattern of lung cancer risk was also the same as that observed in our previous study in this cohort where lung cancer rates from the U.S. population served as an external comparison (Laden et al. 2007). In that report, the SMRs among all drivers and combination workers (SMR = 1.10; 95% CI, 1.02–1.19) and dock-workers (SMR = 1.10; 95% CI, 0.94–1.30) were elevated, whereas for mechanics and hostlers the SMRs were not increased, and for clerks the SMR was statistically significantly decreased. Obtaining similar results using two different approaches in our cohort indicates that the findings are robust.

A greater lung cancer risk in truck drivers has previously been attributed to diesel exhaust because diesel engines manufactured before 2007 in the United States had greater mass emissions of respirable particles with an EC core and adsorbed organic and other PAH compounds than did spark ignition sources. Although spark-ignition light-duty vehicles had lower-mass PM emissions per mile, a much greater number of cars on roadways relative to trucks suggest that automobiles do contribute to vehicle-exhaust–related emissions (Allen et al. 2001; Geller et al. 2005; Zhu et al. 2002). The particle size distributions from gasoline and diesel vehicles are similar, with the ultrafine mode accounting for most particles, and have a similar chemical composition (Kittelson et al. 2003; Kleeman et al. 2000). However, in contrast to pre-2007 diesel engines, particles emitted from the gasoline-powered vehicles are predominantly composed of organic compounds (Kleeman et al. 2000). Under a light load and when idling, diesel emissions from pre-2007 diesel engines also have lower EC and higher OC in PM emissions compared with operating with greater load (Kweon et al. 2002; Shah et al. 2004; Zielinska et al. 2004a, 2004b).

Therefore, as part of our previous exposure assessment (Davis et al. 2006, 2007; Smith et al. 2006), we measured the 2001–2005 terminal background, loading dock, and LH and P&D truck cab EC and OC levels. The EC geometric means (SDs) were as follows: 0.31 (3.72) μg/m^3^ in terminal offices where the clerks worked; 1.12 (1.91) μg/m^3^ in LH drivers; 1.09 (2.48) μg/m^3^ in P&D drivers; 0.76 (2.13) μg/m^3^ in dockworkers; 0.88 (3.04) μg/m^3^ in hostlers; and 2.00 (3.82) μg/m^3^ in mechanics. OC showed less variation, with levels ranging from 19.26 (2.30) μg/m^3^ in LH drivers to 12.40 (1.54) μg/m^3^ in P&D drivers. EC and OC for combination workers would represent a mix of dockworker and P&D driver exposures. We also used structural equation modeling to assess the contribution of background and terminal work area exposures (i.e., on the loading dock) to personal measurements of EC and OC (Davis et al. 2006). Distance from an interstate highway and factors indicating greater freight activity at a terminal were significantly associated with greater personal exposure to EC and OC. Determinants of in-cab exposure to EC and OC in LH and P&D drivers were assessed, and was positively related to ambient particle concentrations attributable to background air pollution and regional traffic (Davis et al. 2007). Given these observations and our exposure measurements, our results suggest that exposure of the drivers comes predominantly from surrounding vehicles and from background air pollution, as well as some exposure related to the driver’s own vehicle, and that loading-dock exposures are related to both background and work area activities.

To identify specific sources of current occupational particulate exposures, we conducted source apportionment studies using particle-phase organic molecular markers in work area samples from seven freight terminals sampled in 2002 and 2003 (Sheesley et al. 2008b) as well as in personal and work area samples in terminal workers and drivers at one terminal (Sheesley et al. 2008a). Source apportionment data in the LH drivers, P&D drivers, and dockworkers indicated that most (~ 80% or greater) of the EC was from diesel sources with a smaller percentage from spark-ignition vehicles and lubricating oil (Sheesley et al. 2008a). OC source apportionment indicated a substantial mobile source contribution that was mostly attributable to lubricating oil. Detailed studies characterizing specific emissions from propane-powered forklift trucks have not been conducted. However, based on measurements obtained in other settings, propane engines produce ultrafine particles (Guo et al. 2004; Rundell 2003) that includes little EC compared with gasoline and diesel engines (Zaebst et al. 1991). Taken together, our results indicate that a variety of mobile sources contribute to ambient vehicle exhaust particles in the trucking industry, with EC representing mainly diesel sources and OC representing a mixture of mobile sources that depend on the work environment.

Our observations regarding lung cancer mortality in truck drivers are supported by a previous case–control study conducted by Steenland et al. (1990) and a companion exposure assessment (1988–1989) (Steenland et al. 1992; Zaebst et al. 1991). They identified cases and controls from retirement records of teamsters who died during 1982–1983 using the Teamster Central States Pension Fund database and obtained smoking history from next of kin. In contrast to our study, where actual work records were available, these researchers obtained work history from next-of-kin report and from self-report from each worker’s retirement application. Although they observed an elevation in smoking-adjusted lung cancer odds ratios for LH and P&D drivers and a positive trend in lung cancer risk with greater duration of work in LH drivers, the risk estimates were imprecise because the population was relatively small. As we have found in our present exposure assessment, geometric mean EC exposures in these late 1980s measurements were similar for LH and P&D drivers (3.8 and 4.0 μg/m^3^, respectively) and approximately 4-fold greater than in the same jobs in our 2001–2005 exposure assessment (Smith et al. 2006). Measurements made in the late 1980s also indicated that exposures in dockworkers were greater than our present exposures and varied by forklift type, with much higher levels associated with diesel and gasoline forklifts (Steenland et al. 1992; Zaebst et al. 1991). However, in contrast to our present results, there was no increase in lung cancer risk among dockworkers in the Steenland et al. study. Unlike our present study, they included relatively few dockworkers, and the category included dispatchers, clerks, and other workers who were unlikely to spend considerable time on a loading dock and would have had little exposure.

Our results are also consistent with a large body of literature indicating a relationship between diesel exhaust exposure and lung cancer risk that have included other groups of drivers (Bhatia et al. 1998; Diesel Working Group 1995; Lipsett and Campleman 1999; Office of Research and Development 2002), and studies in U.S. railroad workers performed by our group (Garshick et al. 2004; Laden et al. 2006a). Our results are also consistent with the results of population-based studies where relationships have been observed between particulate air pollution (Laden et al. 2006b; Pope et al. 1995) or traffic-related expoures (Nafstad et al. 2003; Nyberg et al. 2000) and lung cancer.

The lung cancer risk in the mechanics was not elevated, although they had the highest EC values historically (Zaebst et al. 1991) and in our exposure assessment. Despite present EC values intermediate to dockworkers and drivers, the mortality risk for hostlers was also not increased. Although the sampling plan did not include a detailed assessment of real-time exposures, our field observations indicate that because truck engines are shut off while in the shop area, mechanics are exposed primarily to aged exhaust and only intermittently to fresh exhaust. In contrast, drivers and dockworkers commonly experience periods of more or less continuous exposure to mobile-source related fresh PM related to vehicle exhaust. Hostlers drive primarily small, specialized tractor units in the terminal yard moving trailers to and from the freight dock. Because yard-related truck activity varies throughout each shift, it is also possible that their intermittent pattern of vehicle exhaust exposure accounts for lack of increased risk. One of the potential mechanisms whereby traffic-related exposures may result in DNA damage is due to short-lived reactive oxygen species (ROS). ROS activity has been found to be associated with the smallest particle size fractions and with traffic-related PM compared with other sources, potentially explaining the greater risk in persons in jobs with more constant exposures (Baulig et al. 2004; Briede et al. 2005; de Kok et al. 2006; Li et al. 2003). In addition, the cohort included few hostlers and mechanics, resulting in imprecise effect estimates, and we were unable to fully assess their risk by modeling years of work.

A limitation of this analysis is lack of personal information on potential risk factors for lung cancer. Although cigarette smoking is a major risk factor, the degree to which it is a confounder in this study depends on differences in smoking behavior associated with job title within the cohort. To minimize the possible effect of uncontrolled confounding by smoking, we have studied workers of similar socioeconomic class, a known correlate of smoking habits (Brackbill et al. 1988; Stellman et al. 1988). Although we were unable to directly survey workers in this cohort, we used a representative sample of active and retired workers in the industry (Jain et al. 2006) and found that variation in lung cancer risk among exposed workers did not reflect differences in smoking rates, and adjustment did not significantly influence the observed results other than slightly reducing the effect among LH drivers. These findings are consistent with a recent review of the literature where differences in smoking rates did not explain increased lung cancer risks associated with occupational exposures (Blair et al. 2007). As expected (Thun et al. 1997), we found that smoking rates in the survey varied by age and birth cohort (Laden et al. 2007). Although a limitation of this indirect adjustment is that smoking patterns between jobs may have been different historically, we found that birth-cohort–specific smoking rates were similar among drivers and nondrivers (Laden et al. 2007), suggesting that smoking rates did not vary substantially among jobs. In addition, we tightly controlled all analyses for age and calendar year. It is also unlikely that variation in other potential risk factors for lung cancer (e.g., family history or history of obstructive lung disease) would vary systematically by job title and contribute to confounding.

Another limitation is the lack of job history information before a worker joined one of the four unionized companies in the cohort. The average age of starting work in the trucking industry from the questionnaire was 25 years, which suggests that some workers may have had up to 10 additional years of exposure to mobile-source–related combustion PM in trucking industry jobs. This would reduce the estimated risk attributable to each year of work in Table 3.

## Conclusion

Our results suggest that lung cancer mortality in workers with a previous history of regular exposures to particulate from diesel exhaust and other mobile sources is elevated and increases with increasing exposure duration. The increase in lung cancer risk suggests a contribution from diesel exhaust and a mix of vehicle emissions from other sources because each group of workers had different patterns of current and historical exposures. These results along with previous studies support current efforts to reduce emissions from both diesel vehicles and other sources of vehicle and traffic-related emissions.

## Figures and Tables

**Table 1:** Description of major job categories and work locations of men ≥40 years of age (*n* = 31,135).

Job in 1985	Job duties	No. (%)
Long-haul driver, highway truck cab	Operates heavy duty tractor-trailer trucks between cities	10,825 (35)
Pickup/delivery driver, in and out of truck cab	Drives tractors or smaller single-bodied trucks within cities and rural areas; picks up/delivers cargo between terminal and consumer	5,866 (19)
Dockworker, loading dock	Loads and unload cargo, may operate forklifts	5,710 (18)
Combination worker, truck cab or loading dock,	Combination job: performs activities of either P&D driver or dockworker	4,938 (16)
Mechanic, truck repair shop	Repairs and maintains tractors; job may include fueling	1,741 (6)
Hostler, terminal yard	Drives tractor or “yard tug” (small, specialized tractor units) moving trailers to and from the freight dock and in terminal yard	666 (2)
Clerks, terminal office	Cashiers, dock clerks, dispatchers, customer service representatives, and others not regularly near diesel vehicles	843 (3)
Other jobs	Janitor, unionized manager, trainee, job not defined	546 (2)

**Table 2:** Characteristics of the 31,135 male workers ≥ 40 years of age in 1985.

Characteristic	LH (*n* = 10,825)	P&D (*n* = 5,866)	Combination (*n* = 4,938)	Dock (*n* = 5,710)	Mechanic (*n* = 1,741)	Hostler (*n* = 666)	Clerk (*n* = 843)	Total^a^ (*n* = 31,135)
Age at hire (mean ± SD)	37.5 ± 8.0	35.1 ± 8.5	36.4 ± 8.3	35.3 ± 8.2	34.6 ± 8.2	34.1 ± 8.0	33.2 ± 8.9	36.0 ± 8.3
Age in 1985 (mean ± SD)	49.9 ± 6.1	49.3 ± 6.1	47.8 ± 5.3	48.4 ± 6.0	49.4 ± 6.4	47.7 ± 5.3	49.4 ± 6.4	49.1 ± 6.0
Years of work (mean ± SD)	21.5 ± 8.2	21.1 ± 9.3	21.7 ± 8.3	21.1 ± 8.9	23.5 ± 8.2	23.9 ± 7.4	24.1 ± 10.0	21.6 ± 8.7
Race [*n* (%)]								
Caucasian	9,556 (88.3)	4,799 (81.8)	4,222 (85.5)	4,575 (80.1)	1,463 (84.0)	579 (86.9)	769 (91.2)	26,430 (84.9)
African American	882 (8.1)	519 (8.8)	483 (9.8)	644 (11.3)	171 (9.8)	56 (8.4)	16 (1.9)	2,818 (9.1)
Other/unknown	387 (3.6)	548 (9.3)	233 (4.7)	491(8.6)	107 (6.1)	31 (4.7)	58 (6.9)	1,887 (6.1)
Census region [*n* (%)]								
Northeast	1,050 (9.7)	835 (14.2)	1,006 (20.4)	1,191 (20.9)	206 (11.8)	121 (18.2)	79 (9.4)	4,555 (14.6)
South	4,275 (39.5)	2,135 (36.4)	1,994 (40.4)	1,833 (32.1)	703 (40.4)	223 (33.5)	270 (32.0)	11,682 (37.5)
Midwest	3,754 (34.7)	1,734 (29.6)	1,356 (27.5)	1,834 (32.1)	504 (28.9)	230 (34.5)	252 (29.9)	9,818 (31.5)
West	1,633 (15.1)	1,152 (19.6)	451 (9.1)	807 (14.1)	312 (17.9)	90 (13.5)	239 (28.4)	4,759 (15.3)
Unknown	113 (1.0)	10 (0.2)	131 (2.7)	45 (0.8)	16 (0.9)	2 (0.3)	3 (0.4)	321 (1.0)
Decade of hire [*n* (%)]								
< 1960	883 (8.2)	736 (12.5)	396 (8.0)	521 (9.1)	255 (14.6)	58 (8.7)	150 (17.8)	3,107 (10.0)
1960–1969	2,587 (23.9)	1,661 (28.3)	1,207 (24.4)	1,590 (27.8)	537 (30.8)	214 (32.1)	291 (34.5)	8,284 (26.6)
1970–1979	5,415 (50.0)	2,489 (42.4)	1,821 (36.9)	2,532 (44.3)	712 (40.9)	281 (42.2)	290 (34.4)	13,745 (44.2)
≥ 1980	1,940 (17.9)	980 (16.7)	1,514 (30.7)	1,067 (18.7)	237 (13.6)	113 (17.0)	112 (13.3)	5,999 (19.3)

a Includes the category (*n* = 546) of other workers (janitor, unionized mangers, trainees), which is not shown in the table.

**Table 3:** Lung cancer HRs and 95% CIs associated with ≥ 1 year of work in each job, with mortality assessed 1985 through 2000.

Job title	Person-years	Lung cancer deaths	HR (95% CI)^a^
LH	161,503	323	1.15 (0.92–1.43)
P&D	139,054	233	1.19 (0.99–1.42)
Dockworker	147,513	205	1.30 (1.07–1.58)
Combination	96,543	150	1.40 (1.12–1.73)
Mechanic	25,523	38	0.95 (0.66–1.38)
Hostler	29,947	29	0.99 (0.68–1.45)
Clerk	24,728	15	0.55 (0.32–0.95)
Other jobs	13,040	12	0.89 (0.48–1.63)

a We calculated HRs using regression coefficients from a multivariate Cox proportional hazards regression model with baseline hazards based on age in 1985, decade of hire, and calendar time, with risk sets by attained age, adjusted for the healthy worker survivor effect (total years on work, years off of work), race, and census region.

**Table 4:** Lung cancer mortality percent change per year of work and HRs (95% CIs) associated with cumulative years of work as an LH driver, P&D driver, dockworker, or combination worker, with mortality assessed 1985–2000, and adjustment for estimated differences in smoking behavior.

					HR for 20 years of work	
Job title	Person-years	Lung cancer deaths	Percent change per year of work	Smoking adjustment factor^a^	Multivariate^b^	Smoking adjusted^c^
LH	161,503	323	2.5 (0.2–4.9)	1.17	1.65 (1.04–2.62)	1.40 (0.88–2.24)
P&D	139,054	233	3.6 (1.2–6.1)	0.92	2.04 (1.28–3.25)	2.21 (1.38–3.52)
Dockworker	147,513	205	3.4 (0.8–6.0)	0.96	1.94 (1.18–3.18)	2.02 (1.23–3.33)
Combination	96,543	150	4.0 (1.5–6.6)	0.94	2.20 (1.35–3.61)	2.34 (1.42–3.83)

a Job-specific smoking adjustment factor was calculated by dividing the smoking weighted risk for each job by the smoking weighted risk for all workers not employed in that job.

b HRs were calculated using regression coefficients from multivariate Cox proportional hazard regression models with baseline hazards based on age in 1985, decade of hire, and calendar time, with risk sets by attained age, adjusted for the healthy worker survivor effect (total years on work, years off of work), race, and census region. For ≥ 1 year as ever employed as a clerk, hostler, mechanic, or in an other job. ^c^Smoking-adjusted RR calculated by dividing by the appropriate smoking adjustment factor. 95% CIs were calculated by considering the sampling error in calculating each job-specific correction factor (see “Materials and Methods”).
